# Nutritional status and functions in children with cerebral palsy at different gestational ages

**DOI:** 10.1002/ped4.70080

**Published:** 2026-08-11

**Authors:** Hongmei Tang, Qihong Wu, You Wang, Tingting Peng, Yiting Zhao, Jie Luo, Yaodan Liang, Kaiyin Cai, Linlin Wu, Qinyan Wu, Hongyu Zhou, Yiming Qiu, Jinling Li, Lu He, Kaishou Xu

**Affiliations:** ^1^ Department of Rehabilitation Guangzhou Women and Children's Medical Center Guangzhou Medical University Guangzhou Guangdong China; ^2^ Guangzhou Medical University Guangzhou Guangdong China

**Keywords:** Cerebral palsy, Function, Gestational age, Nutrition, Preterm

## Abstract

**Importance:**

Malnutrition is highly prevalent in children with cerebral palsy (CP) and adversely affects rehabilitation outcomes. Preterm birth is a major risk factor for CP, yet the relationship between functional status and malnutrition in preterm children with CP remains unclear.

**Objective:**

To investigate the effect of gestational age on the association between nutritional status and function levels in children with CP.

**Methods:**

This study included 575 children with CP enrolled between July 2020 and December 2021. Participants were stratified into four groups by gestational age at birth: <28, 28–32, 33–36, and ≥37 weeks. Nutritional status was assessed using weight‐for‐age, weight‐for‐height, height‐for‐age, and body mass index–for‐age *z*‐scores. Gross motor function, eating/drinking ability, and activities of daily living (ADL) were evaluated using Gross Motor Function Classification System (GMFCS), Eating And Drinking Ability Classification System (EDACS), and ADL scales, respectively.

**Results:**

Of the 575 children, 49.0% were born preterm, and 40.4% were categorized as malnutrition. Nutritional status was significantly correlated with gestational age (Kendall's *Tau*‐*b* = 0.13, *P *< 0.01) and all functional measures (*P *< 0.01). Gestational age was correlated with ADL level (Kendall's *Tau*‐*b* = 0.11, *P* < 0.01). Stratified analyses revealed significant correlations between nutritional status and GMFCS, EDACS, and ADL levels in both the 28–32‐week and ≥37‐week groups (all *P* < 0.01). Multinomial logistic regression analysis further demonstrated that gestational age <32 weeks was independently associated with moderate undernutrition (<28 weeks: OR: 4.61, 95% CI: 1.83–11.63, *P* = 0.001; 28–32 weeks: OR: 2.71, 95% CI: 1.56–4.72, *P* < 0.001).

**Interpretation:**

Nutritional status in preterm children with CP, particularly those born before 32 weeks of gestation, correlates significantly with motor function, feeding ability, and daily living skills. These findings may inform the development of individualized rehabilitation strategies for this high‐risk population.

## Graphical abstract

The nutritional status of cerebral palsy was significantly associated with their motor, eating and drinking ability, and their ability of daily living in the 28–32‐week and the ≥37‐week gestational age group, whereas in 33–36‐week group, correlation was only observed between the nutritional status and eating and drinking ability.

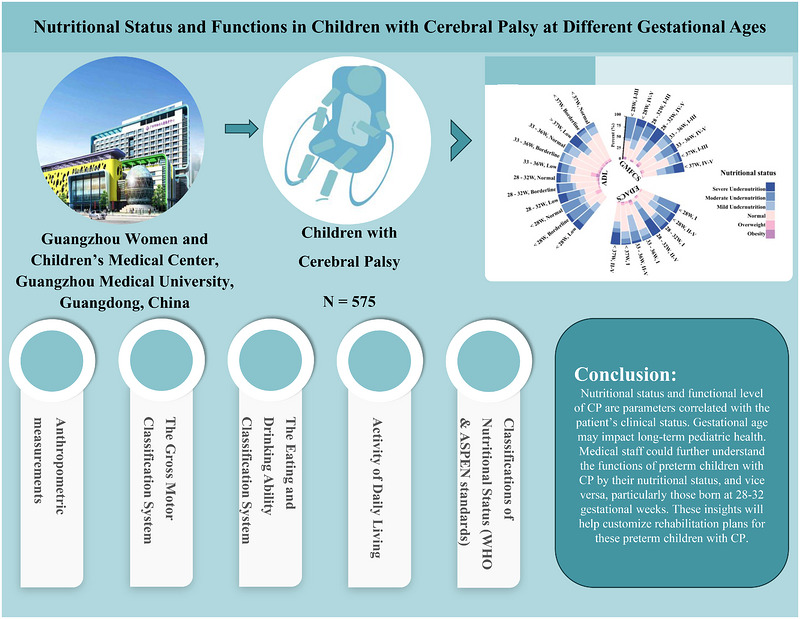

## INTRODUCTION

Cerebral palsy (CP) is a permanent disorder of the central motor and postural development in fetuses or infants, characterized by static abnormalities in the developing brain, with a global prevalence of 1.6‰–3.4‰.^1^, ^2^ Its etiology is multifactorial, involving genetic mutations, intrauterine infections, abnormal fetal inflammation, and preterm delivery.^3^, ^4^, ^5^ The associated conditions in children with CP include intellectual disability, epilepsy, drooling, feeding difficulties, gastroesophageal reflux, and malnutrition. One risk factor for CP is preterm birth, which mediates half of the pre‐existing maternal conditions and pregnancy complications.^3^

The nutritional status of children with CP has become a focus of clinical concern. Studies show that 46%–76.6% of children with CP suffer from malnutrition,^6^, ^7^ whereas feeding and swallowing problems occur in approximately 50%.^8^ Motor function in children with CP is also associated with their nutritional status. Progressive deterioration of motor function and feeding ability contributes to malnutrition.^9^, ^10^, ^11^ Furthermore, poorer nutritional status has been inversely associated with neurodevelopmental outcomes in infants at high risk of CP.^12^ This evidence suggests a potential vicious cycle between CP and malnutrition, in which each factor exacerbates the other. Therefore, assessing the nutritional status of children with CP is indispensable for optimizing rehabilitation outcomes and supporting physical and mental development.

Preterm delivery is a major risk factor for CP, with the incidence rising as gestational age decreases.^13^ The underlying neuropathology may also differ with gestational age due to variations in fetal growth across developmental periods. It is therefore essential to analyze the characteristics of children with CP based on stratified gestational age. However, the relationship between function and malnutrition in preterm children with CP remains unclear. The present study aimed to explore how nutritional status correlates with function in a cohort of extremely preterm, very preterm, late preterm, and term‐born children with CP to help physicians and therapists better understand these children's physical conditions and tailor treatment strategies.

## METHODS

### Ethical approval

This study was approved by the Health Research Committee of Guangzhou Women and Children's Medical Center (2020‐29601) and registered on the Chinese Clinical Trial Registry (ChiCTR2000033869). All children's family members signed informed consent forms.

### Study design and participants

This cross‐sectional study included 575 children with CP who underwent rehabilitation and follow‐up at the outpatient service of the Rehabilitation Department of the Women and Children's Medical Center, Guangzhou Medical University in Guangzhou, China, between July 2020 and December 2021. Figure S1 shows the flow chart of this study.

Inclusion criteria comprised a diagnosis of CP based on international diagnostic criteria issued in 2007,^14^ age between 1 and 18 years. Malnutrition in children under 5 years of age was diagnosed according to the WHO growth charts and the consensus of the American Society for Parenteral and Enteral Nutrition (ASPEN), whereas ASPEN only was used for the older cohort (6–18 years).^15^, ^16^

Exclusion criteria included degenerative neurological diseases, congenital heart disease, acute or chronic hepatitis, malignant tumors, or uncontrollable epilepsy. Additionally, children with inherited metabolic disorders affecting growth and development, including Down syndrome, Rett syndrome, and Angelman syndrome, were also excluded.

Participants were categorized into four groups based on their gestational age: <28, 28–32, 33–36, and ≥37  weeks. No enrolled children received nutritional intervention.

### Anthropometric measurements

Anthropometric measurements are commonly used, accessible, and cost‐effective assessments that directly reflect the nutritional status of children with CP. Anthropometric measurements were conducted by trained therapists using standardized methods during their initial visit. In this study, several parameters were measured, including height (>2 years)/body length (1–2 years old), and weight. For children under 5 years of age, head circumference (HC), mid‐upper arm circumference (MUAC), triceps skinfold thickness (TSF), and subscapular skinfold thickness (SSF) were also recorded and calculated using WHO Anthro software (version 3.2.2). The classification of HC followed WHO standards (*z*‐score cutoff of −2 and −3).^17^ MUAC, TSF, and SSF were assessed according to the ESPGHAN using the 10th percentile (P10) red flag criterion.^18^ Additionally, MUAC was also distinguished based on the criteria from our previous work on children with CP to further assess their nutritional status.^19^ For children with CP aged <12 years who were unable to stand or had spasms, height (cm) was estimated using Stevenson's equation [height (cm) = (tibial length × 3.26) + 30.8].^20^ Chumlea's equation was used for children with CP aged ≥12 years.^21^ Children's birth weights were also recorded.

### Nutritional status assessment

The *z*‐score is an internationally recognized standard for assessing the growth and nutritional status of children. Trained therapists conducted the assessments at the time of enrollment. The nutritional status of the participants was classified as mild, moderate, or severe undernutrition, overweight, obesity, or normal according to the WHO growth standards, and the ASPEN standards were incorporated to refine the distinctions between moderate and mild undernutrition. This assessment comprised four components: weight‐for‐age *z*‐score (WAZ), weight‐for‐height *z*‐score (WHZ), height‐for‐age *z*‐score (HAZ), and body mass index (BMI)‐for‐age *z*‐score. The diagnostic criteria for undernutrition were set as mild (−2 ≤ WHZ or WAZ < −1), moderate (−3 ≤ WHZ, WAZ, or HAZ < −2), and severe (WHZ, WAZ, or HAZ < −3).^15^, ^16^, ^22^ The BMI *z*‐score was used to define obesity (BMI *z*‐score > 2) and overweight (BMI *z*‐score > 1).^23^ To further distinguish children with CP who were constitutionally small but adequately nourished, red flag warning signs from ESPGHAN were adopted.^18^

### Assessment of motor function and functional activities

The gross motor function of children with CP was classified by trained therapists using the Gross Motor Function Classification System (GMFCS) into five levels: Levels I–II, walking independently; Level III, walking with an assistive device; and Levels IV–V indicate disability of walking.^24^ Fundamental differences exist in functional independence and care needs. To highlight independent mobility and ensure model stability, GMFCS was collapsed into a binary variable: Levels I–III (ambulatory) and Levels IV–V (non‑ambulatory). The Eating and Drinking Ability Classification System (EDACS) was used to classify eating and drinking abilities into five levels, where Level I indicates safe and efficient eating, Level II indicates inefficient but safe eating, and Levels III–V indicate limitations in eating safety or inability to eat safely.^25^ Levels II–V all represent feeding disorders. To clarify the impact of feeding dysfunction on outcomes and to mitigate the risk of overfitting due to small subgroup samples, EDACS was dichotomized into two categories: no impairment (Level I) and impairment (Levels II–V). Abilities in activities of daily living (ADL) were assessed using the Social Life Ability Scale for Infant Junior Middle School Student, and each child's parents or guardians completed the assessment after the evaluators explained each item. All evaluations were performed at the children's initial visit. Finally, the scales’ raw scores were converted to normalized scores for different age groups, ranging from extremely low to mildly below average (normalized scores of 5–8), borderline (normalized score of 9), and normal to very good (normalized scores of 10–13).^26^

### Statistical analysis

To minimize potential bias in data interpretation, all analyses were performed by researchers who were not involved in recruitment and had no contact with children. Data analysis and the development of multinomial logistic regression equations were conducted using SPSS version 26.0 (IBM Corp.) and R version 4.3.3. Categorical variables were presented as frequencies and percentages. The relationships between dichotomous variables of motor function (GMFCS levels I–III vs. IV–V), eating and drinking ability (EDACS Levels I vs. II–V), and ordered categorical variables of gestational age and nutritional status, were assessed using Kendall's *Tau*‐*b* correlation analysis to test for associations and linear trends. Spearman correlation analysis was performed to assess the relationship between continuous variables of birth weight and ordinal categorical variables. A cross‐tabulation of nutritional status and the other functional variables by gestational age was also conducted. Kendall's *Tau*‐*b* correlation analysis was performed within each stratum. Bonferroni‐adjusted *P* values were used to evaluate the significance of the correlations. For regression, only the primary effect (gestational age) remains interpreted at the unadjusted *α* = 0.05. As all independent variables were categorical, the linearity assumption in the logit was not applicable. Multicollinearity was assessed using the variance inflation factor (VIF), with all values below 5.71. Statistical significance was set at *P* < 0.05.

## RESULTS

### Participants’ characteristics

A total of 575 children were enrolled in this study (381 males and 194 females), with a mean age of 3.7 ± 2.9 years (range 1–17 years) between July 2020 and December 2021. There were 421 children under 5 years of age and 154 older children. All the enrolled children from the outpatient clinic were fed orally. Spastic CP comprised 94.3% of all the CP types. Less than half of the participants were born preterm (gestational age <37 weeks) (49.0%) and had a low birth weight (<2.5 kg) (47.1%) (Table 1). Furthermore, a total of 40.3% of the children were categorized as malnutrition. And more than half had limitations in performing daily activities (borderline, 28.4%; below average, 29.4%). Table 1 presents the demographic and clinical characteristics of the participants. Data for MUAC, HC, TSF, and SSF were unavailable for 27 of the 421 children under 5 years of age for multiple reasons (Tables S1–S3). Their nutritional risk across gestational age groups was assessed using ESPGHAN red flag warning signs, results were shown in Table S4. Additionally, three cross‐tabulations of malnutrition classification by gestational age were presented, based on different criteria: MUAC for nutrition screening, WHO and ASPEN standards, and WHO and ASPEN and ESPGHAN standards (Tables S5–S7).

**TABLE 1:** Demographic and clinical characteristics of children with cerebral palsy

Variables	Total (*n* = 575)
Age at data collection (years)	
1–≤3	260 (45.2)
3–≤6	229 (39.8)
6–≤12	72 (12.5)
12–≤18	14 (2.4)
Gestational age (weeks)	
<28	34 (5.9)
28–32	162 (28.2)
33–36	86 (14.9)
≥37	293 (51.0)
Birth weight (kg)	
<2.5	271 (47.1)
≥2.5	304 (52.9)
Type of cerebral palsy	
Spastic	542 (94.3)
Dystonia	20 (3.5)
Ataxic	4 (0.7)
Mixed	9 (1.5)
GMFCS level	
I–III	486 (84.5)
IV–V	89 (15.5)
EDACS level	
I	465 (80.9)
II–V	110 (19.1)
ADL	
Normal	243 (42.3)
Borderline	163 (28.3)
Low	169 (29.4)
Nutritional status	
Severe undernutrition	48 (8.3)
Moderate undernutrition	97 (16.9)
Mild undernutrition	87 (15.1)
Normal	304 (52.9)
Overweight	26 (4.5)
Obesity	13 (2.3)

*Note*: Data were shown as *n* (%).

Abbreviations: ADL, activities of daily living; EDACS, Eating and Drinking Ability Classification System; GMFCS, Gross Motor Function Classification System.

### Analysis of gestational age and nutritional status

**FIGURE 1:**
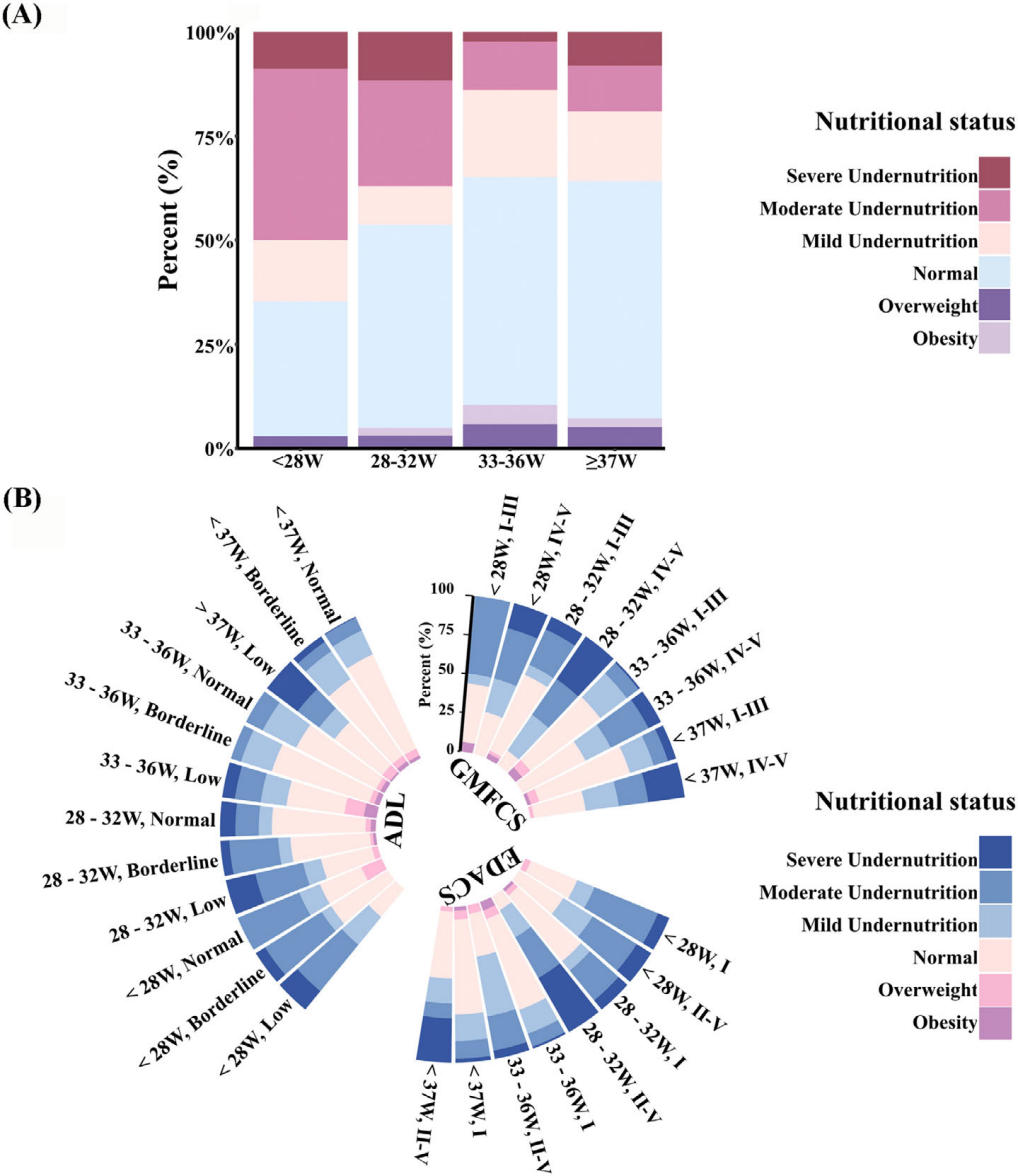
Relationship between nutritional status and gestational age in enrolled children with CP. (A) The distributions of each nutritional status of children with CP, grouped by gestational age. (B) The proportion of each nutritional status in different GMFCS, EDACS, and ADL levels, stratified by gestational age. ADL, activities of daily living; CP, cerebral palsy; EDACS, Eating and Drinking Ability Classification System; GMFCS, Gross Motor Function Classification System.

Over half of the enrolled children (51.0%) were born at or after 37 weeks of gestation. More than one‐quarter (28.2%) were born between 28 and 32 weeks of gestation, whereas only 5.9% were born before 28 gestational weeks. Less than half of the children exhibited an abnormal nutritional status. Mild, moderate, and severe undernutrition accounted for 15.1%, 16.9%, and 8.3%, respectively. Overweight and obesity accounted for only 6.8% of the total participants (Table 1). The distribution of nutritional status of each group is shown in Figure 1A. In the 28–32, 33–36, and ≥37‐week gestational age groups, children with normal nutritional status were the most prevalent, whereas moderate undernutrition was most common in those born at <28 weeks. Compared to the ≥37‐week gestational age group, children with CP who were born before 28 gestational weeks had a 3.18 times higher probability of undernutrition (95% CI: 1.48, 6.83; *P* < 0.05), whereas those who were born between 28 and 32 weeks of gestation had a 1.51 times increased risk (95% CI: 1.01, 2.25; *P* < 0.05) (Table 2). However, similar probabilities of malnutrition were observed between the ≥37‐ and 33–36‐week gestational age groups.

**TABLE 2:** Analysis of malnutrition in children with cerebral palsy by groups of gestational age

Gestational age (weeks)	Normal nutrition, *n* (%)	Undernutrition			Overweight and Obesity		
		*n* (%)	OR (95% CI)	*P*	*n* (%)	OR (95% CI)	*P*
<28	11 (32.4)	22 (64.7)	3.18 (1.48–6.83)	<0.05	1 (2.9)	0.72 (0.09–5.89)	0.76
28–32	79 (48.8)	75 (46.3)	1.51 (1.01–2.25)	<0.05	8 (4.9)	0.81 (0.34–1.90)	0.62
33–36	47 (54.7)	30 (34.9)	1.02 (0.60–1.71)	0.96	9 (10.5)	1.52 (0.65–3.55)	0.33
≥37	167 (57.0)	105 (35.8)	–	–	21 (7.2)	–	–

Abbreviations: CI, confidence interval; OR, odd ratio.

### Correlation analysis of gestational age, nutritional status, and the functional status of CP

To explore relationships among nutritional status, gestational age, and the functional status of CP, pairwise correlation analyses were conducted. As shown in Table 3, nutritional status correlated positively with birth weight, gross motor function, eating and drinking ability, and ADL performance (*P *< 0.01), with the strongest correlation observed between nutritional status and GMFCS level (Kendall's *Tau*‐*b* = 0.40). Additionally, the gestational age was positively correlated with nutritional status, birth weight, and ADL (*P* < 0.01). In contrast, no correlation was found between gestational age and GMFCS level (*P* = 0.08), or EDACS level (*P* = 0.14).

**TABLE 3:** Correlation analysis of nutritional status, GMFCS, EDACS, ADL, birth weight and gestational age

Variables	Birth weight		Nutritional status		GMFCS		EDACS		ADL	
	*R*	*P*	Kendall's *Tau*‐*b*	*P*	Kendall's *Tau*‐*b*	*P*	Kendall's *Tau*‐*b*	*P*	Kendall's *Tau*‐*b*	*P*
Gestational age	0.73	<0.01	0.13	<0.01	0.08	0.08	−0.06	0.14	0.11	<0.01
Nutritional status	0.24	<0.01	–	–	0.40	<0.01	0.24	<0.01	0.21	<0.01

Abbreviations: ADL, activities of daily living; EDACS, Eating and Drinking Ability Classification System; GMFCS, Gross Motor Function Classification System.

To further investigate these relationships, analyses stratified by gestational age were performed. The distribution of nutritional status across gestational ages and ratings is illustrated in Figure 1B. Table 4 details the correlations of nutritional status with GMFCS, EDACS, and ADL levels within each group of gestational age. In groups with a gestational age of 28–32 weeks and ≥37 weeks, significant correlations were found between nutritional status and both GMFCS (*P* < 0.01) and ADL level (*P* < 0.01). In groups of 28–32 (*P* < 0.01), 33–36 (*P* = 0.01), and ≥37 weeks (*P* < 0.01) of gestational age, EDACS level was significantly correlated with nutritional status. No correlations were observed between nutritional status and either GMFCS or ADL level in groups with a gestational age of <28 and 33–36 weeks.

**TABLE 4:** Kendall *Tau*‐*b* analysis of GMFCS, EDACS, and ADL with nutritional status stratified by gestational age

	GMFCS				EDACS				ADL				
Gestational age (week)	IV–V, *n* (%)	I–III, *n* (%)	Kendall's *Tau*‐*b*	*P*	II–V, *n* (%)	I, *n* (%)	Kendall's *Tau*‐*b*	*P*	Below average, *n* (%)	Borderline, *n* (%)	Normal, *n* (%)	Kendall's *Tau*‐*b*	*P*
<28													
Severe	3 (100.0)	0 (0.0)	0.15	0.33	1 (33.3)	2 (66.7)	0.00	1.00	0 (0.0)	1 (33.3)	2 (66.7)	0.17	0.23
Moderate	6 (42.9)	8 (57.1)			3 (21.4)	11 (78.6)			3 (21.4)	6 (42.9)	5 (35.7)		
Mild	4 (80.0)	1 (20.0)			2 (40.0)	3 (60.0)			1 (20.0)	1 (20.0)	3 (60.0)		
Normal	5 (45.5)	6 (54.6)			3 (27.3)	8 (72.7)			2 (18.2)	6 (54.6)	3 (27.3)		
Overweight	0 (0.0)	1 (100.0)			0 (0.0)	1 (100.0)			1 (100.0)	0 (0.0)	0 (0.0)		
Obesity	0 (0.0)	0 (0.0)			0 (0.0)	0 (0.0)			0 (0.0)	0 (0.0)	0 (0.0)		
28–32													
Severe	6 (31.6)	13 (68.4)	0.27	<0.01	8 (42.1)	11 (57.9)	0.32	<0.01	6 (31.6)	3 (15.8)	10 (52.6)	0.21	<0.01
Moderate	5 (12.2)	36 (87.8)			6 (14.6)	35 (85.4)			9 (22.0)	16 (39.0)	16 (39.0)		
Mild	5 (33.3)	10 (66.7)			4 (26.7)	11 (73.3)			5 (33.3)	4 (26.7)	6 (40.0)		
Normal	2 (2.5)	77 (97.5)			2 (2.5)	77 (97.5)			36 (45.6)	26 (32.9)	17 (21.5)		
Overweight	0 (0.0)	5 (100.0)			0 (0.0)	5 (100.0)			2 (40.0)	1 (20.0)	2 (40.0)		
Obesity	0 (0.0)	3 (100.0)			0 (0.0)	3 (100.0)			2 (66.7)	1 (33.3)	0 (0.0)		
33–36													
Severe	1 (50.0)	1 (50.0)	0.20	0.06	1 (50.0)	1 (50.0)	0.30	0.01	0 (0.0)	0 (0.0)	2 (100.0)	−0.01	0.90
Moderate	3 (30.0)	7 (70.0)			4 (40.0)	6 (60.0)			4 (40.0)	2 (20.0)	4 (40.0)		
Mild	2 (11.1)	16 (88.9)			7 (38.9)	11 (61.1)			8 (44.4)	6 (33.3)	4 (22.2)		
Normal	5 (10.6)	42 (89.4)			5 (10.6)	42 (89.4)			19 (40.4)	19 (40.4)	9 (19.2)		
Overweight	0 (0.0)	5 (100.0)			1 (20.0)	4 (80.0)			1 (20.0)	1 (20.0)	3 (60.0)		
Obesity	0 (0.0)	4 (100.0)			0 (0.0)	4 (100.0)			1 (25.0)	1 (25.0)	2 (50.0)		
>37													
Severe	10 (41.7)	14 (58.3)	0.25	<0.01	18 (75.0)	6 (25.0)	0.24	<0.01	1 (4.2)	3 (12.5)	20 (83.3)	0.23	<0.01
Moderate	8 (25.0)	24 (75.0)			6 (18.8)	26 (81.3)			13 (40.6)	5 (15.6)	14 (43.8)		
Mild	9 (18.4)	40 (81.6)			10 (20.4)	39 (79.6)			24 (49.0)	15 (30.6)	10 (20.4)		
Normal	14 (8.4)	153 (91.6)			27 (16.2)	140 (83.8)			95 (56.9)	41 (24.6)	31 (18.6)		
Overweight	1 (6.7)	14 (93.3)			2 (13.3)	13 (86.7)			7 (46.7)	3 (20.0)	5 (33.3)		
Obesity	0 (0.0)	6 (100.0)			0 (0.0)	6 (100.0)			3 (50.0)	2 (33.3)	1 (16.7)		

### Multinomial logistic regression analysis of the determinants of nutritional status

Multinomial logistic regression was employed due to the violations of the assumptions of multiple linear regression. The analysis (Table 5) revealed that no parameters significantly influenced the probability of overweight or obesity in these CP children. For undernutrition, the severity of ADL impairment was positively associated with the probabilities of both moderate undernutrition (OR = 1.55, 95% CI: 1.12–2.13, *P* = 0.008) and severe undernutrition (OR = 2.34, 95% CI: 1.40–3.91, *P* = 0.001). In contrast, the severity of EDACS impairment was positively associated solely with severe undernutrition (OR = 5.17, 95% CI: 2.26–11.81, *P* < 0.001). Notably, compared to children with CP born at term, those born at <28 weeks and 28–32 weeks of gestational age had OR of 4.61 (95% CI: 1.83–11.63, *P* < 0.001) and 2.71 (95% CI: 1.56–4.72, *P* < 0.001), respectively, for moderate undernutrition.

**TABLE 5:** Regression analysis of factors associated with nutritional status

Variable	Obesity		Overweight		Mild undernutrition		Moderate undernutrition		Severe undernutrition	
	OR (95% CI)	*P*	OR (95% CI)	*P*	OR (95% CI)	*P*	OR (95% CI)	*P*	OR (95% CI)	*P*
Gestational age (weeks)										
≥37	ref	–	ref	–	ref	–	ref	–	ref	–
33–36	2.01 (0.53–7.64)	0.307	1.08 (0.37–3.16)	0.892	1.30 (0.69–2.49)	0.421	1.02 (0.49–2.25)	0.970	0.23 (0.05–1.08)	0.062
28–32	0.84 (0.20–3.52)	0.807	0.61 (0.21–1.78)	0.368	0.74 (0.39–1.42)	0.365	2.71 (1.56–4.72)	<0.001	2.10 (0.98–4.49)	0.057
<28	/	/	1.10 (0.13–9.52)	0.930	1.07 (0.33–3.41)	0.916	4.61 (1.83–11.63)	0.001	1.13 (0.26–4.85)	0.874
Sex										
Male	ref	–	ref	–	ref	–	ref	–	ref	–
Female	0.16 (0.02–1.24)	0.079	0.94 (0.40–2.19)	0.880	1.02 (0.62–1.69)	0.934	0.83 (0.49–1.38)	0.46	0.39 (0.17–0.88)	0.024
GMFCS	/	0.998	0.27 (0.03–2.30)	0.232	2.59 (1.19–5.67)	0.017	1.73 (0.82–3.68)	0.153	1.94 (0.81–4.69)	0.139
EDACS	/	0.998	0.76 (0.20–2.90)	0.690	1.92 (0.97–3.81)	0.063	1.21 (0.59–2.45)	0.601	5.17 (2.26–11.81)	<0.001
ADL	1.39 (0.68–2.85)	0.369	1.72 (1.02–2.88)	0.041	0.89 (0.63–1.27)	0.530	1.55 (1.12–2.13)	0.008	2.34 (1.40–3.91)	0.001

Abbreviations: ADL, activities of daily living; CI, confidence interval; EDACS, Eating and Drinking Ability Classification System; GMFCS, Gross Motor Function Classification System; OR, odd ratio.

## DISCUSSION

To our knowledge, this is the first study to investigate the nutritional status and function of children with CP stratified by gestational age. Our findings suggest that the nutritional status of children with CP correlates with their level of functional impairment at various gestational ages.

Preterm birth is a critical risk factor for CP, with the risk increasing as gestational age decreases.^27^ We therefore performed analyses to examine the relationship between gestational age and functional outcomes in children with CP. Notably, a significant relationship was observed between gestational age and birth weight in preterm infants, who were often of low birth weight and at risk of poor nutritional status. However, no significant relationship was observed between gestational age and motor functional status or eating and drinking ability in children with CP. These findings align with previous studies that report no significant association between gestational age and the level of GMFCS.^28^ It is noteworthy that the incidence of CP decreases with advancing gestational age.^28^ In other words, the severity of neurological impairment likely depends on factors beyond gestational age alone.

Notably, preterm birth is also a probable contributor to malnutrition, which is a common complication in children with CP. This may be because of two reasons. First, the establishment of a normal fetal swallowing‐breathing rhythm begins at 28 weeks and matures at 32–34 weeks. Thus, preterm infants, especially those born before 32 weeks, frequently exhibit weak sucking, poor chewing, dysphagia, and subsequent malnutrition. This study also demonstrated a positive correlation between the nutritional status of children with CP and their EDACS level. This study innovatively analyzed their relationship based on stratified gestational age, indicating that enhanced eating and drinking ability may be associated with better nutritional outcomes in children with CP who were born after 28 gestational weeks, consistent with our hypothesis. Second, dysphagia in preterm infants with CP leads to prolonged feeding times and inadequate nutritional intake, which may be associated with concurrent central nervous system impairments. Insufficient energy intake therefore impacts the nutritional status of preterm children with CP. Approximately half of children with CP experience feeding (50%; 95% CI 36.0%–64.8%) and swallowing difficulties (53%; 95% CI 40.7%–65.9%; moderate certainty).^2^ In our cohort, 19.1% of children with CP experienced dietary problems, risking deficiencies in iron, zinc, and other nutrients, whereas loss of appetite further exacerbates malnutrition. Thus, this study further supports early interventions for children with CP and dysphagia, including postural management, food texture adjustment, oral sensorimotor training, botulinum toxin injection, and neuromuscular electrical stimulation.^29^ For children with CP at risk of unsafe feeding, enteral nutrition should be considered to ensure adequate energy intake and reduce the risk of aspiration.^18^ Consequently, the gestational age may affect nutritional status through the maturation of swallowing–breathing rhythms, the mode of nutritional intake, and the extent of neurological damage.

A multicenter study from Argentina suggested children with CP at GMFCS IV–V had a 4‐fold risk of malnutrition compared to those at GMFCS I–III, with a 14‐fold increase in the likelihood of severe undernutrition.^9^ This is consistent with our observation of a positive correlation between malnutrition and GMFCS level. One explanation is that an elevated GMFCS level reflects more intense muscle cramps, increased involuntary movements, and reduced postural control in children with CP. These factors likely elevate their energy expenditure. In addition, preterm birth often causes feeding difficulties and dysphagia, resulting in insufficient nutrient intake and exacerbating malnutrition. Our results indicate that children in the groups of 28–32 and ≥37 gestational weeks may be more responsive to improvements in gross motor function following nutritional enhancement, and vice versa. A similar trend can also be seen in the relationship between nutritional status and the ADL level. Very preterm delivery persistently disrupts cortical development,^30^ suggesting that preterm delivery and associated nervous system impairment may complicate the physical condition of children born before 28 weeks of gestation. This likely involves intrinsic factors that are not rescued easily by self‐healing and daily care, obscuring a clear relationship. Consequently, more comprehensive indicators and early interventions are needed. Hence, children with both CP and malnutrition, regardless of functional severity, should receive not only nutritional interventions, such as high‐energy formula feeds,^22^ but also effective rehabilitation therapies to promote their comprehensive function, which probably play an important role in improving their nutritional status.

Our previous works also examined the relationship between nutritional status and multiple functions in children with CP. This study broadened the spectrum of parameters analyzed beyond the work of Zhao et al.,^10^ which primarily assessed the relationship between nutritional status and GMFCS, EDACS, respectively. Furthermore, it provides a more substantial evidence base for refinement of nutritional formulas to address undernutrition and gross motor dysfunction in children with CP.^22^

However, this study has several limitations. First, the age range of the enrolled children was broad. Enrolled children may have been exposed to additional confounding factors, such as socioeconomic status and physical environment, which could influence the results. Though all included children were fed orally and receiving rehabilitation at the Women and Children's Medical Center, children likely came from diverse cultural backgrounds with varying dietary habits. Feeding method was classified simply as oral or tube feeding, and children reliant on tube feeding were excluded. In outpatient cohorts, patients reliant on tube feeding were inadvertently excluded. Consequently, this limits the generalizability of the findings to severe populations. Based on 2017 ESPGHAN guidelines,^18^ 3.0% of children with CP were identified as constitutionally small, but adequately nourished. Besides, 40.7% of CP children classified as normal nutritional status showed one or more red flag warning signs. Further data were needed to assess the utility of the classification system combined with WHO, ASPEN, and ESPGHAN. Additionally, although our model was pre‐specified based on clinical rationale to test a single primary hypothesis, the exploratory nature of the functional covariates (GMFCS, EDACS, and ADL) warrants cautious interpretation and validation in future studies. To advance, a prospective cohort study adhering to the ESPGHAN 2017 guidelines is critically needed in the future. Furthermore, information on maternal conditions during pregnancy was unavailable, which may provide insights into the association between gestational age and nutritional status in children with CP.

In conclusion, nutritional status and functional level are correlated parameters in the clinical assessment of children with CP. Gestational age influences long‐term pediatric health. Children with CP born before 32 weeks of gestation are more likely to experience moderate or severe undernutrition. Medical staff could infer the function of preterm children with CP by their nutritional status, and vice versa, particularly those born at 28–32 gestational weeks. These findings may be generalizable to similar populations, given our use of established methodologies and the observation of comparable phenomena in other studies. Such insights will help tailor rehabilitation plans for preterm children with CP. Consequently, the active development of early nutritional interventions is urgent to improve rehabilitation outcomes, whereas early rehabilitation training may concurrently promote their nutritional status.

## CONFLICT OF INTEREST

The authors declare no conflicts of interest.

## Supporting information



Supporting Information
