# Supplementary material for: Nutritional status and functions in children with cerebral palsy at different gestational ages
Source: Pediatr Investig. 2026 Aug 11;10(5):385–94. doi: 10.1002/ped4.70080 (PMC13460276; doi:10.1002/ped4.70080)
Supplement: Supplementary file 1 [file PED4-10-385-s001.pdf]

# Supplementary Material for

**Nutritional status and functions in children with cerebral palsy at different gestational ages**

Hongmei Tang, Qihong Wu, You Wang, Tingting Peng, Yiting Zhao, Jie Luo, Yaodan Liang, Kaiyin Cai, Linlin Wu, Qinyan Wu, Hongyu Zhou, Yiming Qiu, Jinling Li, Lu He, Kaishou Xu

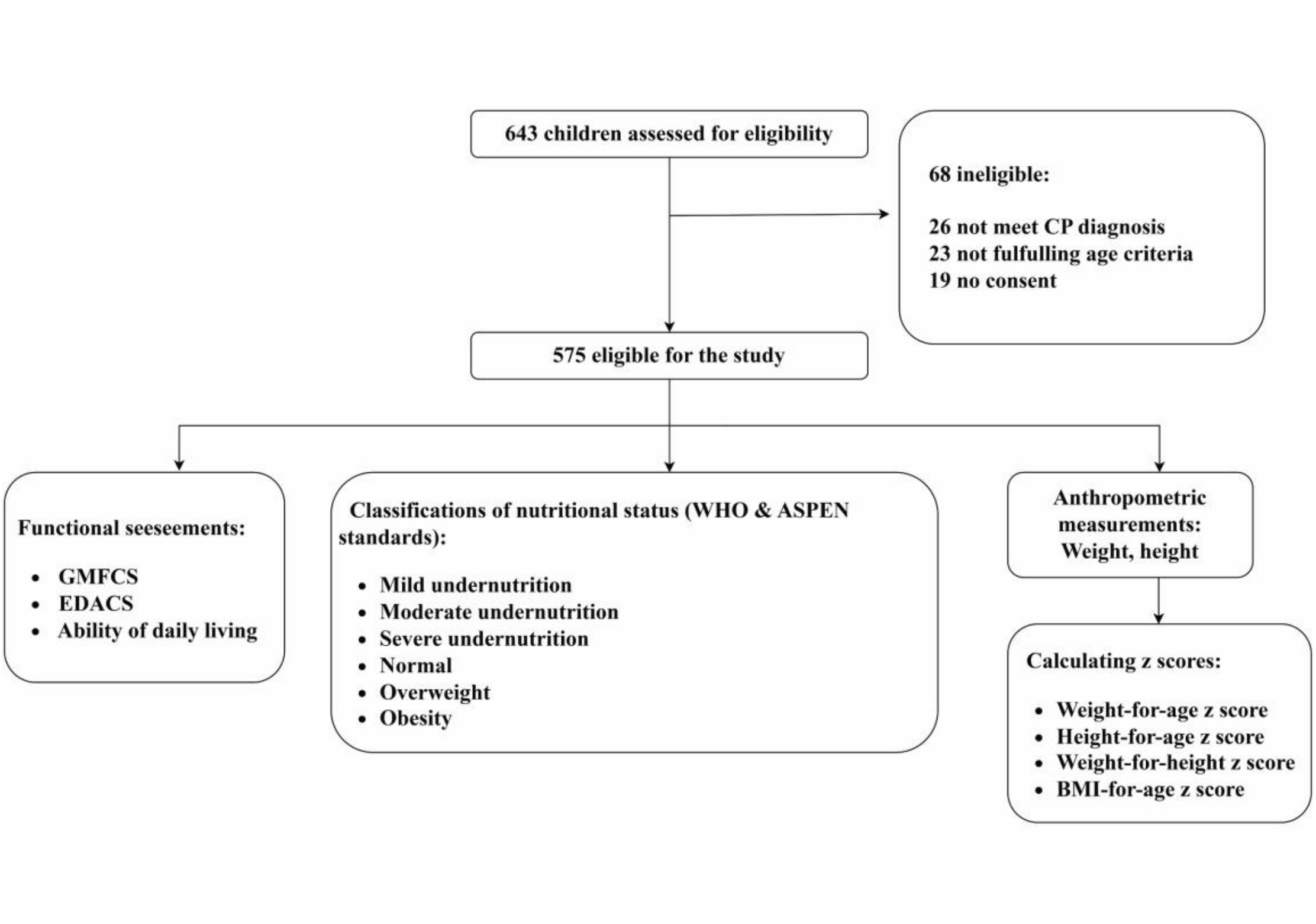


**Figure S1.** Flow chart of the overall research. CP, cerebral palsy; GMFCS, Gross Motor Function Classification System; EDACS, Eating and Drinking Ability Classification System; ADL, Activities of Daily Living; WHO, World Health Organization; ASPEN, American Society for Parenteral and Enteral Nutrition; BMI, body mass index.

**Table S1.** Children with cerebral palsy stratified by Z score of head circumference range and gestational age ($n$ = 394)

| Gestational age (weeks) | Z score of Head Circumference $n$ (%) | | | | |
|---|---|---|---|---|---|
| | Z < -3 | -3 ≤ Z < -2 | -2 ≤ Z ≤ 2 | Z > 2 | total |
| <28 | 4 (17.39) | 3 (13.04) | 16 (69.57) | / | 23 |
| 28-32 | 10 (8.93) | 17 (15.18) | 85 (75.89) | / | 112 |
| 33-36 | 3 (5.26) | 6 (10.53) | 48 (84.21) | / | 57 |
| ≥37 | 30 (14.85) | 23 (11.39) | 147 (72.77) | 2 (0.99) | 202 |
| Total | 47 (11.93) | 49 (12.44) | 296 (75.13) | 2 (0.51) | 394 |

**Table S2.** Distribution of children with cerebral palsy above and below the 10th percentile of TSF and SSF by gestational Age ($n$ = 394)

| **Gestational age (weeks)** | **TSF Status Relative to 10th Percentile** | | | **SSF Status Relative to 10th Percentile** | | |
|---|---|---|---|---|---|---|
| | ***n* (%)** | | | ***n* (%)** | | |
| | **< P10** | **≥ P10** | **total** | **< P10** | **≥ P10** | **total** |
| <28 | 10 (43.48) | 13 (56.52) | 23 | 5 (21.74) | 18 (78.26) | 23 |
| 28-32 | 35 (31.25) | 77 (68.75) | 112 | 39 (34.82) | 73 (65.18) | 112 |
| 33-36 | 23 (40.35) | 34 (59.65) | 57 | 20 (35.09) | 37 (64.91) | 57 |
| ≥37 | 65 (32.18) | 137 (67.82) | 202 | 73 (36.14) | 129 (63.86) | 202 |
| total | 133 (33.76) | 261 (66.24) | 394 | 137 (34.77) | 257 (65.23) | 394 |

TSF: triceps skinfold thickness; SSF: subscapular skinfold thickness.

**Table S3.** Distribution of children with cerebral palsy above and below the 10th percentile of MUAC by gestational age ($n$ = 394)

| Gestational age (weeks) | MUAC Status Relative to 10th Percentile *n* (%) | | |
|---|---|---|---|
| | **< P10** | **≥ P10** | **total** |
| <28 | 3 (13.04) | 20 (86.96) | 23 |
| 28-32 | 6 (5.36) | 106 (94.64) | 112 |
| 33-36 | 4 (7.02) | 53 (92.98) | 57 |
| ≥37 | 26 (12.87) | 176 (87.13) | 202 |
| Total | 39 (9.90) | 355 (90.10) | 394 |

MUAC: Mid-upper arm circumference.

**Table S4.** Nutritional risk stratification using ESPGHAN Red Flag Warning Signs across gestational age groups in children with cerebral palsy (*n* = 394)

| **Gestational age (weeks)** | **Nutritional Status (with Red Flag Warning Signs from ESPGHAN) *n* (%)** | | | | | |
|---|---|---|---|---|---|---|
| | **Normal** | **Overweight / Obesity** | **Normal with Potential Risk** | **Undernutrition** | **Constitutionally Small, Adequately Nourished** | **total** |
| <28 | 6 (26.09) | / | 4 (17.39) | 13 (56.52) | / | 23 |
| 28-32 | 31 (27.68) | 1 (0.89) | 24 (21.43) | 55 (49.11) | 1 (0.89) | 112 |
| 33-36 | 13 (22.81) | 4 (7.02) | 19 (33.33) | 19 (33.33) | 2 (3.51) | 57 |
| ≥37 | 77 (38.12) | 3 (1.49) | 40 (19.80) | 73 (36.14) | 9 (4.46) | 202 |
| Total | 127 (32.23) | 8 (2.03) | 87 (22.08) | 160 (40.61) | 12 (3.05) | 394 |

**Table S5.** Frequency distribution of nutritional status based on Mid-upper Arm Circumference across gestational age groups (*n* = 394)

| Gestational age (weeks) | Mid-upper Arm Circumference for Nutrition Screening in Outpatient with Cerebral Palsy *n* (%) | | | | | | |
|---|---|---|---|---|---|---|---|
| | severe undernutrition MUAC<14.35cm | moderate undernutrition 14.35cm≤MUAC<15.05cm | mild undernutrition 15.05cm≤MUAC<15.35cm | normal 15.35cm≤MUAC≤17.55cm | overweight 17.55cm<MUAC≤20.40cm | obesity MUAC>20.40cm | total |
| <28 | 1 (4.35) | 6 (26.09) | 2 (8.70) | 10 (43.48) | 3 (13.04) | 1 (4.35) | 23 |
| 28-32 | 16 (14.29) | 25 (22.32) | 7 (6.25) | 51 (45.54) | 12 (10.71) | 1 (0.89) | 112 |
| 33-36 | 7 (12.28) | 15 (26.32) | 8 (14.04) | 16 (28.07) | 8 (14.04) | 3 (5.26) | 57 |
| >=37 | 45 (22.28) | 39 (19.31) | 10 (4.95) | 86 (42.57) | 19 (9.41) | 3 (1.49) | 202 |
| Total | 69 (17.51) | 85 (21.57) | 27 (6.85) | 163 (41.37) | 42 (10.66) | 8 (2.03) | 394 |

MUAC: Mid-upper Arm Circumference

**Table S6.** Frequency distribution of nutritional status based on WHO & ASPEN across gestational age groups (*n* = 394)

| Gestational age (weeks) | WHO & ASPEN standards for Nutrition Screening in Outpatient with Cerebral Palsy *n* (%) | | | | | | |
|---|---|---|---|---|---|---|---|
| | severe undernutrition | moderate undernutrition | mild undernutrition | normal | overweight | obesity | total |
| <28 | 2 (8.70) | 9 (39.13) | 2 (8.70) | 10 (43.48) | 0 (0.00) | 0 (0.00) | 23 |
| 28-32 | 15 (13.39) | 28 (25.00) | 13 (11.61) | 55 (49.11) | 1 (0.89) | 0 (0.00) | 112 |
| 33-36 | 0 (0.00) | 7 (12.28) | 14 (24.56) | 32 (56.14) | 2 (3.51) | 2 (3.51) | 57 |
| >=37 | 23 (11.39) | 20 (9.90) | 39 (19.31) | 117 (57.92) | 2 (0.99) | 1 (0.50) | 202 |
| Total | 40 (10.15) | 64 (16.24) | 68 (17.26) | 214 (54.31) | 5 (1.27) | 3 (0.76) | 394 |

**Table S7.** Frequency distribution of nutritional status based on WHO & ASPEN & ESPGHAN across gestational age groups (*n* = 394)

| Gestational age (weeks) | WHO & ASPEN & ESPGHAN standards for Nutrition Screening in Outpatient with Cerebral Palsy | | | | | | |
|---|---|---|---|---|---|---|---|
| | *n* (%) | | | | | | |
| | severe undernutrition | moderate undernutrition | mild undernutrition | normal | overweight | obesity | total |
| <28 | 2 (8.70) | 7 (30.43) | 2 (8.70) | 12 (52.17) | 0 (0.00) | 0 (0.00) | 23 |
| 28-32 | 8 (7.14) | 16 (14.29) | 24 (21.43) | 63 (56.25) | 1 (0.89) | 0 (0.00) | 112 |
| 33-36 | 0 (0.00) | 4 (7.02) | 24 (42.11) | 25 (43.86) | 2 (3.51) | 2 (3.51) | 57 |
| >=37 | 21 (10.40) | 11 (5.45) | 64 (31.68) | 103 (50.99) | 2 (0.99) | 1 (0.50) | 202 |
| Total | 31 (7.87) | 38 (9.64) | 114 (28.93) | 203 (51.52) | 5 (1.27) | 3 (0.76) | 394 |
